# Use of a Novel Extremophilic Xylanase for an Environmentally Friendly Industrial Bleaching of Kraft Pulps

**DOI:** 10.3390/ijms232113423

**Published:** 2022-11-03

**Authors:** Nazaré Almeida, Valérie Meyer, Auphélia Burnet, Jeremy Boucher, David Talens-Perales, Susana Pereira, Petri Ihalainen, Thomas Levée, Julio Polaina, Michel Petit-Conil, Susana Camarero, Paula Pinto

**Affiliations:** 1 RAIZ—Forest and Paper Research Institute, Rua José Estevão 221, Eixo, 3800-783 Aveiro, Portugal; 2 Centre Technique du Papier—The French Pulp and Paper Technical Centre, Domaine Universitaire, CS90251, CEDEX 9, 38044 Grenoble, France; 3 Fibre Excellence Saint-Gaudens—Rue du President Saragat, 31800 Saint-Gaudens, France; 4 Instituto de Agroquímica y Tecnología de Alimentos, CSIC Agustín Escardino 7, 46980 Paterna, Valencia, Spain; 5 MetGen Oy, Rakentajantie 26, 20780 Kaarina, Finland; 6 Centro de Investigaciones Biológicas Margarita Salas, CSIC, Ramiro de Maeztu 9, 28040 Madrid, Spain

**Keywords:** extremophilic xylanase, kraft pulp, ECF bleaching, chlorine dioxide savings

## Abstract

Xylanases can boost pulp bleachability in Elemental Chlorine Free (ECF) processes, but their industrial implementation for producing bleached kraft pulps is not straightforward. It requires enzymes to be active and stable at the extreme conditions of alkalinity and high temperature typical of this industrial process; most commercial enzymes are unable to withstand these conditions. In this work, a novel highly thermo and alkaline-tolerant xylanase from *Pseudothermotoga thermarum* was overproduced in *E. coli* and tested as a bleaching booster of hardwood kraft pulps to save chlorine dioxide (ClO_2_) during ECF bleaching. The extremozyme-stage (EXZ) was carried out at 90 °C and pH 10.5 and optimised at lab scale on an industrial oxygen-delignified eucalyptus pulp, enabling us to save 15% ClO_2_ to reach the mill brightness, and with no detrimental effect on paper properties. Then, the EXZ-assisted bleaching sequence was validated at pilot scale under industrial conditions, achieving 25% ClO_2_ savings and reducing the generation of organochlorinated compounds (AOX) by 18%, while maintaining pulp quality and papermaking properties. Technology reproducibility was confirmed with another industrial kraft pulp from a mix of hardwoods. The new enzymatic technology constitutes a realistic step towards environmentally friendly production of kraft pulps through industrial integration of biotechnology.

## 1. Introduction

Global concern about climate change has led authorities and governments to implement strategies and rules to address the protection of the environment and natural habitats. In line with the 2030 Agenda for Sustainable Development, the European Green Deal encompasses a set of measures for sustainable growth towards a circular economy, such as more restrictive laws against polluting processes and the investment in environmentally friendly technologies to force industries to make a transition towards cleaner and greener processes. Even before this, the pulp and paper industry had already undergone a major transformation towards the development of cleaner processes in the past few decades, particularly in the pulp bleaching plant. The concern about formation of chlorinated dioxins and other chlorinated compounds during pulp bleaching resulted in a technological revolution in the pulp industry. However, the commitment to remove elemental chlorine (responsible for the formation of chlorinated dioxins) from pulp production in ECF pulps, and thereafter to eliminate chlorine-derived reagents (i.e., chlorine dioxide) in the so-called totally chlorine free (TCF) processes, requires the development and optimisation of new bleaching sequences [1,2,3,4]. In this endeavour, biotechnology can significantly help with the transition to greener pulp production industrial processes. The use of different types of enzymes as biocatalysts to aid pulp bleaching and delignification has been widely reported [5,6,7]. Laccases and xylanases are the preferred biocatalysts used as “prebleaching” or “bleaching boosters” to improve pulp bleachability. For instance, xylanase treatment of pulp after oxygen delignification has been shown to be effective in some ECF sequences [5,6,7,8], allowing fewer bleaching chemicals to be used, which has economic benefits.

During kraft pulping, short xylan chains precipitate at the surface of the fibres acting as a physical barrier against the action of the bleaching agents [9], leading to substantial consumption of ClO_2_. Pretreatment of pulp with xylanases boosts the action of the bleaching chemicals by breaking the xylans and subsequently reducing the concentration of long xylan chains on the fibre surface, which increases their permeability [10]. Nevertheless, for the feasible integration of an enzymatic stage in the industrial process, the enzyme must be active and stable at the operating industrial conditions. This is particularly relevant for the kraft process, given the harsh physicochemical conditions that characterise this pulping method, which is used predominantly by the paper industry to obtain pulp from wood. Most of the commercial enzymes are unable to withstand the conditions of high temperatures and alkaline pH used in this particular industry [11].

The great diversity of microorganisms capable of colonising diverse habitats in nature constitutes many potential sources of enzymes for industry. In particular, extremophilic bacteria or archaea adapted to extreme temperatures, pH, pressure or salinity become valuable sources of enzymes to be applied as biocatalysts at the harsh operational conditions of the industrial processes [12]. In silico screening of protein sequences from databases, often derived from genomic and metagenomic analysis, has been successfully applied to identify extremophilic enzymes [11,13,14].

A bioinformatic screening of the glycosyl hydrolase family GH10 allowed the identification and characterisation of xylanases active at high pH and temperature. One enzyme in particular, Xyn11, a xylanase from *Pseudothermotoga thermarum*, showed an exceptional xylanolytic activity at 90 °C and pH 10.5 [11]. Here, this extremophilic enzyme (extremozyme) is integrated in an industrial ECF bleaching sequence of eucalyptus kraft pulp with the aim of increasing pulp bleachability and reducing the need for ClO_2_ as bleaching agent. After optimising the conditions of the extremozyme stage at laboratory scale, the developed extremozyme-aided sequence was validated at pilot scale.

## 2. Results and Discussion

**Figure 1:**
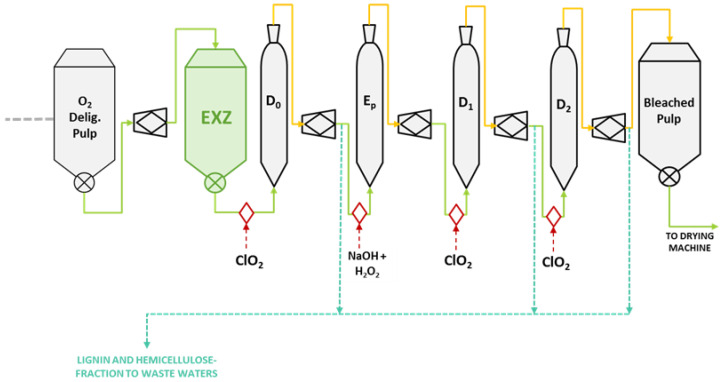
Scheme of the typical production process of a bleached kraft pulp (grey) with enzymatic treatment, EXZ stage (green), applied after the O_2_ delignification stage.

The novel extremophilic xylanase Xyn11 from *Pseudothermotoga thermarum*, with max. activity at pH 10.5 and 90 °C [11], was assayed as a pretreatment stage (EXZ) of industrial oxygen-delignified eucalyptus kraft pulp with the objective of saving ClO_2_, and compared with the conventional industrial bleaching sequence (D_0_E_p_D_1_D_2_) (Figure 1). Three different sets of experiments were carried out to evaluate the effect of introducing an enzymatic stage in the bleaching process and to optimise a xylanase-aided bleaching sequence: EXP (1) optimisation of the duration of the enzymatic stage at the optimal conditions for the activity of the enzyme; EXP (2) reduction in the amount of ClO_2_ required for bleaching the pulp; and EXP (3) evaluation of different extremophilic xylanases.

### 2.1. Effect of Xyn11 Pretreatment Duration (EXP 1)

**Figure 2:**
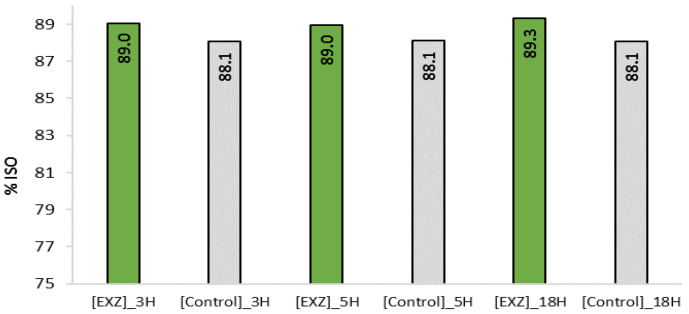
Final pulp brightness (after D_2_) obtained through Xyn11 pretreatment for 3, 5 and 18 h, followed by a full bleaching sequence, compared to the corresponding control sequences.

Considering the influence of the reaction time on xylanase effect [7], pulp was treated with Xyn11 during 18 h, 5 h or 3 h. Comparing the final brightness (after D_2_) of the resulting pulps, the best result (89.3% ISO) was obtained for the pulp bleached after 18 h of enzymatic treatment (Figure 2). After 3 and 5 h, the final brightness was 0.49 and 0.38% ISO points lower, respectively. The enzymatic prebleaching of the pulp exerted a positive impact on pulp brightness, with gains from 0.77 to 1.25 ISO points, compared to the control pulps, which showed a final brightness of 88.1% ISO. However, since 18 h of enzymatic treatment is not compatible with mill scale trials, 5 h of reaction was selected to proceed with the next experiments.

### 2.2. Potential ClO_2_ Savings with Xyn11 Pretreatment (EXP 2)

Given the well reported savings of ClO_2_, by applying an enzymatic pretreatment on pulp [5,6,7,9], three different ClO_2_ loads (reduced by 15%, 20% and 25% with respect to the ClO_2_ required in the conventional sequence) were assayed. The objective was to confirm the good performance of Xyn11 as bleaching enhancer and to evaluate its potential to reduce the need of the bleaching chemicals, while achieving the industrial reference brightness (88.1% ISO).

**Figure 3:**
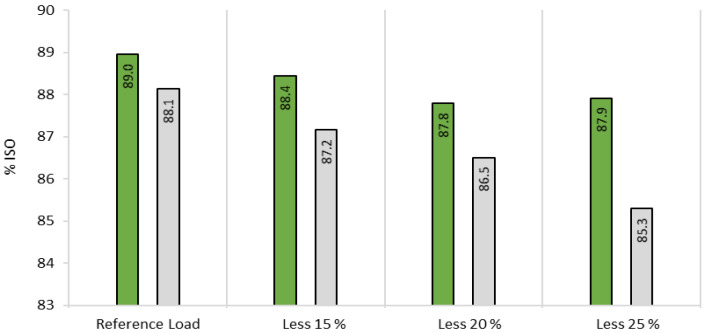
Final pulp brightness (after D_2_) obtained after 5 h prebleaching stage with Xyn11 (green) followed by bleaching with reference ClO_2_ load or with 15%, 20% or 25% less ClO_2_ in each D step, compared with the corresponding controls (grey).

Brightness levels (after D_2_) obtained with the different ClO_2_ loads (Figure 3) showed that 15% of ClO_2_ can be saved to reach a brightness level of 88.4% ISO, higher than the industrial reference, by adding the 5 h-prebleaching stage with Xyn11. The results obtained for the control pulp with 15% less ClO_2_ (87.2% ISO) confirmed the positive effect of the EXZ stage on pulp bleachability.

### 2.3. Comparison between Different Xylanases (EXP3)

**Figure 4:**
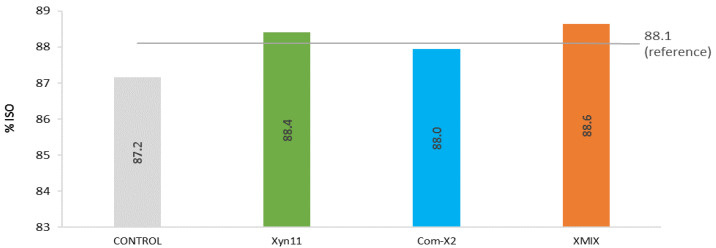
Final pulp brightness obtained after 5 h prebleaching stage with different xylanases followed by 15% less ClO_2_ applied in each D step of the bleaching sequence.

Three xylanases, namely Xyn11, a commercial xylanase (Com-X2) and a mix of both (XMIX), were tested as prebleaching stages of the O_2_-delignified eucalyptus kraft pulp using the optimised conditions: 5 h of enzymatic treatment (EXP1) and 15% less ClO_2_ in D stages (EXP2). All resulting pulps clearly outperformed the control. Treatments with Xyn11 or XMIX allowed us to achieve a brightness level (≥88.4% ISO) higher than the industrial reference (Figure 4).

Papermaking properties were determined to evaluate the effect of the enzymatic stage, and possible differences between the three tested xylanases in terms of the pulp quality. The observed structural properties followed the expected trend; the increase in pulp refining enhanced fibre bonding, leading to a higher drainage index and Gurley air resistance due to the lower amount of water and air (respectively) passing through the fibre network.

**Figure 5:**
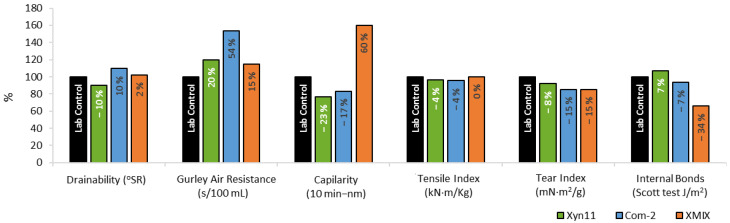
Papermaking properties obtained for pulps pretreated with different xylanases and bleached with 15% less ClO_2_ compared to the control pulp (no xylanase addition) at laboratory scale.

Figure 5 shows the papermaking properties of the enzymatically bleached pulps refined with 2000 revolutions (to obtain a drainage index close to the 30 °SR of the market bleached pulp) compared to that of control pulp (with no ClO_2_ savings and no enzyme addition). In general, pulp properties were not significantly affected by the enzymatic stages (most variations were within the instrumental error). However, Com-X2 and XMIX had more impact on the porosity of the fibre network. The internal bonding, tear index and tensile index were less effected by Xyn11 treatment.

Despite the reported impact of xylanase in the crystallinity index of cellulose [15], previous work (not published) performed by RAIZ with enzymatic bleaching, led to a reduction in pulp hemicelluloses around 0.5% (*w*/*w*), indicating that the crystallinity/amorphous ratio of the pulps should not be affected due the low amount of xylans released. In light of this, the crystalline index of the pulp was determined via X-ray diffraction. No major differences were detected for all the xylanase-treated pulps compared to the control one (Figure S1, Table S1). Thus, it can be inferred that low amounts of xylose were released, as expected.

**Figure 6:**
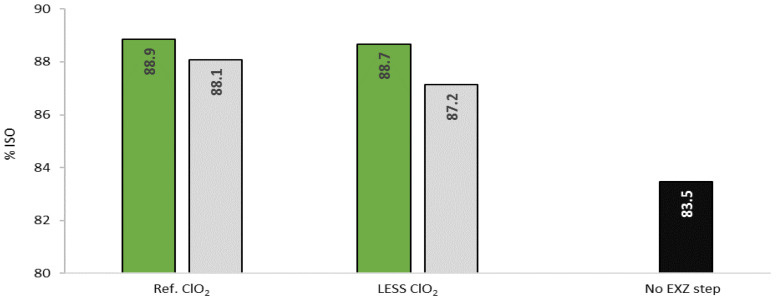
Comparison of pulp brightness obtained after 3 h pretreatment with Xyn11 (green) or without enzyme (control, grey) followed by conventional bleaching sequence applying reference or 15% less ClO_2_ loadings; and with 15% less ClO_2_ but without EXZ step (No EXZ step, black).

Taking into account the good pulp brightness level (89.0% ISO) obtained by 3 h treatment with Xyn11 (Figure 2), and given that Xyn11 stage has no detrimental effect on pulp properties, the possibility of saving ClO_2_ with a shorter enzymatic treatment (3 h instead of 5 h) was evaluated. This trial would enable us to define the best performance of EXZ stage combined with the use of bleaching conditions closest to the mill ones before pilot trial validation. Again, 15% less ClO_2_ was added at each D stage of the bleaching sequence (D_0_E_p_D_1_D_2_). Three hours of enzymatic treatment were enough to exceed (88.7% ISO) the brightness of the mill reference (88.1% ISO), even when 15% less ClO_2_ was used in the bleaching sequence, confirming the bleaching potential of Xyn11 (Figure 6). The trial also demonstrated the positive effect that the extreme conditions of temperature and pH applied have on pulp brightness, even without the addition of enzyme.

### 2.4. Pilot Validation of the Xyn11-Assisted Bleaching of Eucalyptus Kraft Pulp

To validate the laboratory results, the optimised EXZ-assisted bleaching sequence was accomplished at pilot scale on washed O_2_-delignified pulp under relevant industrial conditions. This pulp had a brightness of 57% ISO, a kappa number of 11 and a drainage index of 18 °SR. The reference industrial bleaching sequence increased the brightness of the pulp to 89.0% ISO, with 100% ClO_2_ used. In contrast, the integration of the EXZ stage in the bleaching sequence allowed us to reach 91.1% ISO brightness after D_2_ stage, using 15% less ClO_2_ (Table 1), thus confirming it is possible to save ClO_2_ and outperform the reference industrial brightness. The brightness of the pulp bleached with the Xyn11-assisted sequence after D_1_ stage (90.7% ISO) was already higher than that of the reference mill pulp after D_2_. Therefore, additional ClO_2_ savings are achievable. Indeed, it is possible to save 25% of ClO_2_ by suppressing the final D_2_ bleaching stage in the EXZ-aided bleaching sequence.

The kappa number of the pulp, measured after the extraction stage (E_p_), decreased with the enzymatic pretreatment, suggesting more lignin was released from the fibres even when 15% less ClO_2_ was applied in D_0_ stage. After the Xyn11-aided bleaching sequence, the degree of polymerisation of cellulose (DPv) was significantly higher than that of the reference bleached pulp, indicating that the cellulose is less degraded and that the lignin is more easily extracted from the fibre wall during the D stages. Finally, the drainage index slightly increased with the Xyn11-assisted bleaching sequence, indicating that the refining of this pulp should be adapted and will generate some electrical energy savings (Table 1).

**Figure 7:**
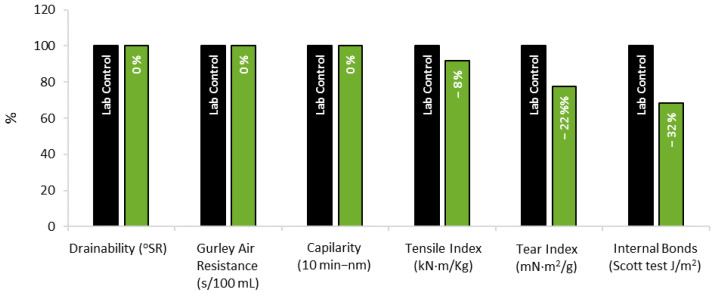
Papermaking properties obtained for the pulp bleached with the Xyn11-assited sequence with 15% ClO_2_ savings (green) in relation to the control (with no xylanase addition) at pilot scale.

Regarding the papermaking properties, similar results were obtained for both enzymatically bleached and reference pulps. The quality of the pulp was not compromised by the enzymatic stage before the bleaching sequence (Figure 7).

The effluents generated by the EXZ-assisted (EXZD_0_EpD_1_) and reference bleaching (D_0_EpD_1_D_2_) sequences were compared. One important issue in the bleaching of chemical pulps is the generation of organochlorinated compounds. As expected, the EXZ stage did not generate absorbable organic halides (AOX) and, due to the reduction in the ClO_2_ charge in each D stage, the total AOX content in the effluent was reduced by 18% at the end of the enzymatic sequence. This 18% global reduction for the whole bleaching effluent was calculated based on the proportion of each bleaching stage contributing to AOX: 6% reduction for D_0_, 0% for Ep and 20% for D_1_ (Table S2). This reduction in AOX makes the bleaching effluent more amendable to secondary wastewater biotreatments, given AOX are bio-recalcitrant and toxic to anaerobic methanogenic bacteria [15], as well as to aerobic aquatic microorganisms having a negative impact on the biodegradability of the organic matter present in the effluents.

Here, the chemical oxygen demand (COD) of the effluent increased after EXZ stage, most probably due to the higher release of hemicellulose-derived sugars (mainly from xylans) and lignin-based compounds by Xyn11 treatment. However, considering the sum from each bleaching stage, the total COD of the effluent was reduced by 44% after the XD_0_E_p_D_1_ sequence (Table S2). The overall biological oxygen demand (BOD) of the effluent from the EXZ-assisted sequence was, however, slightly higher. The higher the BOD/COD ratio, the higher the biodegradability. This indicates that the substances generating the COD are more biodegradable [16]. The EXZ-assisted pulp bleaching generates an effluent which is two times more biodegradable than the control pulp bleaching, reducing the environmental impact and improving the running of the wastewater treatment plant of the mill.

### 2.5. Evaluation of the EXZ-Assisted Bleaching Sequence on Other Hardwood Kraft Pulps

**Figure 8:**
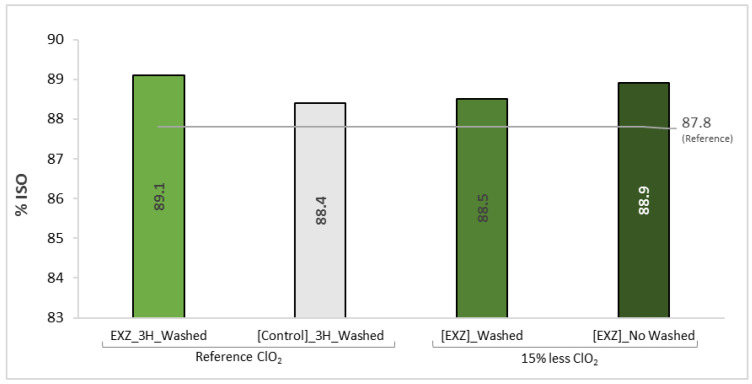
Final brightness of a mixed hardwood pulp obtained after bleaching with the EXZ-bleaching sequence. The reference brightness (87.8% ISO) refers to the pulp usually obtained at mill.

The Xyn11 stage was reproduced on a mixed-hardwoods kraft pulp under the above optimised conditions. The effect of the enzymatic stage was tested on O_2_-delignified pulp with and without washing to evaluate the effect of the residual lignin on the enzyme activity. With the application of the enzymatic treatment before the washing step, it is possible to apply the enzyme directly to the pulp suspension, avoiding the adjustment of the suspension to the optimal pH for the xylanase. Thus, for the trials with less 15% ClO_2_, washed and non-washed pulps were used to evaluate the effect of the original filtrates on Xyn11 performance (Figure 8).

Similar results were obtained for mixed-hardwood kraft pulp as for eucalyptus kraft pulp. The introduction of the Xyn11 stage allowed savings of 15% ClO_2_ in the bleaching sequence. Both enzymatically treated pulps bleached with 15% less ClO_2_ had higher brightness levels than the mill reference (87.8% ISO), suggesting that higher ClO_2_ savings may be possible. The best final brightness was obtained with the unwashed pulp (88.9% ISO).

Other studies reported in the literature about pretreatment of hardwood and/or herbaceous kraft pulps with xylanases have proved to reduce the need for chlorine-based chemicals during pulp bleaching. However, some of these reports entail the combined use of xylanase with other enzyme degradation activities to enhance pulp bleachability and achieve comparable chemical savings to those obtained here [8,17,18,19,20]. In others, xylanase treatment of kraft pulps does not combine alkaline pH and high temperature [21,22]; these conditions are far from those necessary for a viable integration of the enzymatic step in the industrial process. This is the reason why the potential of xylanases with extremophilic properties as bleaching boosters has been indicated in other studies [23,24,25].

Moving forward in this line of investigation, it was demonstrated in this work that the feasible extremozyme-assisted ECF bleaching of hardwood kraft pulps at relevant industrial conditions and pilot scale obtained remarkable environmental benefits and bleaching efficiency gains while maintaining pulp brightness and papermaking properties. In addition, the exceptional alkaliphilic properties and thermophilicity of the novel xylanase Xyn11 allows its direct addition to unwashed O_2_-delignified hardwood pulp. This is a significant factor from the industrial point of view, in terms of minimising changes in the current industrial process for pulp production, as the enzymatic stage is carried out at pH 10.5 and 90 °C.

## 3. Materials and Methods

### 3.1. Raw Material

Industrial O_2_-delignified eucalyptus kraft pulp with 60% ISO brightness and a kappa number around 12 was supplied by a Portuguese mill. This pulp has a brightness close to 60% ISO and a kappa number around 12.

Industrial O_2_-delignified kraft pulp obtained from a mix of hardwoods (poplar, beech, oak and chestnut) was supplied by Fibre Excellence Saint Gaudens mill. This pulp has a 43% ISO brightness and a kappa number of 11. The enzymatic treatment was performed in the O_2_-delignified pulp before and after the washing step. The washing step was carried out on an industrial scale using vapor condensates produced during the cooking process (11 ton of condensates per ton of pulp).

### 3.2. Enzyme Production

The scale up and pilot scale production of Xyn11 was conducted in 400 L BR300 fermentor (Belach Bioteknik AB, Skogås, Sweden) monitored by Biophantom 2000 (Belach Bioteknik AB, Skogås, Sweden) using MetGen’s ENZINE^®^ technology platform and *E. coli* production strain [26]. See Table S3 for medium composition. The pH monitoring and control was performed by EasyFerm Plus PHI S8 120 (Hamilton, Bonaduz, Switzerland) automatic addition of 4 M HCl or 28–30% NH_4_OH. Oxygenation of the cells was maintained by constant aeration and automatic increases in stirring. Dissolved oxygen level (set point: 30%) was monitored throughout the cultivation with Hamilton VisiFerm DO ARC 120 (Hamilton). Fermentation was followed by offline monitoring of OD600, relative activity and base consumption. Product formation during the fermentation was verified by sampling hourly for xylanase activity determination using endo-1,4-β-D-XYLANASE assay with Azo-Xylan substrate (Birchwood) from Megazyme (Wicklow, Ireland) with the following steps: (1) prepare dilution 1:20 in lysis buffer (1×) using whole cell broth; (2) incubate 20 µL of solution with 130 µL of 0.1 M NaPO4 Buffer (pH 6.0); (3) incubate samples for 10 min at 45 °C with 1000 rpm; (4) add 150 µL 1% Azo-Xylan solution and continue incubation for 10 min; (5) stop reaction by adding 600 µL 100% EtOH and cool down on ice for 5 min; (6) spin samples and load 200 µL into 96-well plate and measure absorbance at 590 nm.

After fermentation was completed, cells were lysed according to MetGen’s standard operation protocols. This includes lysing treatment (18 h, at 25 °C) with lysis solution followed by a heat treatment at 60 °C for 30 min. Lysis solution composition is as follows (per volume or weight for 1 L of lysis solution): Triton X-100—100 mL, Citric acid anhydrous—34.8 g, NaOH (pH adjustment to 7.0) and DI water for volume adjustment to 1 L. In total, 273 kg of unconcentrated broth was collected and ultrafiltrated (Alfa Laval PilotUnit Multi equipped with two Koch Membrane Systems, cut-off 10 kDa). Around 50 kg of product was harvested after ultrafiltration. The product quality was verified by measuring pH profile at 85 °C.

### 3.3. Enzymatic Pretreatment

The enzymatic treatment (EXZ) was applied as a prebleaching stage on O_2_-delignified eucalyptus kraft pulp at the optimum conditions of Xyn11 activity, 90 °C and pH 10.5 [11]. The pulp was previously diluted using mill effluent (from O_2_ delignified tower) until it reached a 10% pulp consistency to better reproduce the mill conditions. One ml of Xyn11 was added per 100 g of o.d. pulp at 10% consistency, which represents 5000 units (measured with beechwood xylan at pH 9.0 and 80 °C) per 100 g of pulp.

Next, the pulp was bleached at different stages with a combination of ClO_2_, H_2_O_2_ and NaOH, using the conventional bleaching sequence of the mill (D_0_E_p_D_1_D_2_). Total ClO_2_ used in D-stages varied between 3.0% and 3.2% o.d. pulp, with a total duration of 325 min at 75 °C. The load of H_2_O_2_ and NaOH used on Ep stage varied between 0.44 and 0.66% and 1.22 and 1.77%, respectively. Chemicals loading was adjusted according to the kappa number and brightness of the pulp to process.

### 3.4. Optimisation of the Extremozyme-Bleaching Sequence

Three different sets of experiments were carried out:

EXP 1. The enzymatic treatment was carried out at Xyn11 optimal conditions (90 °C, pH 10.5) during 18 h, 5 h or 3 h at 10% consistency.
EXP 2. This set of experiments were carried out at the best time tested in EXP 1 (5 h) and same conditions, but reducing the amount of ClO_2_ by 15%, 20% or 25% in each D stage. The objective was to find the highest savings in ClO_2_ that allowed reaching the reference pulp brightness obtained with the control test (88.1% ISO).
EXP 3. In this set of experiments Xyn11, Com-X2 (a commercial MetZyme^®^ xylanase provided by MetGen, Kaarina, Finland), and a mixture of both enzymes in a ratio 1:1 *v*/*v* named XMIX were used in the previously optimised conditions: 5 h, 90 °C and pH 10.5 for EXZ stage, and 15% less ClO_2._

### 3.5. X-ray Diffraction

Structural analysis of the pulps was performed using the X-ray diffraction technique (XRD, PANalytical, X’Pert Pro model, Almelo, The Netherlands) in a Bragg-Brentano geometry with CuKα radiation (λ = 1.5406 Å) at 45 KV and 40 mA equipped with X’Celerator detector. The diffractograms were obtained with a range of 0.0501° in an angular amplitude 2θ from 10 to 40°. Crystallinity index values were calculated according to the Segal peak height method [27].

### 3.6. Pilot Scale Trial

The Xyn11 stage was conducted at CTP bleaching pilot plant (Grenoble, France) with 110 kg of o.d. pulp, at the conditions optimised at laboratory scale: 1 mL of Xyn11/100 g of o.d. pulp, 90 °C, 3 h, 10% pulp consistency and initial pH of 10.5. The subsequent D_0_E_p_D_1_D_2_ sequence was applied with the same conditions as for the reference sequence, except the ClO_2_ charge (which is reduced by 15% in each D stage as performed at laboratory scale).

In order to guarantee the chemical integrity and quality of the pulp during the transportation, and considering that the filtrates obtained after oxygen delignification are classified as hazardous substances, which could lead to a considerable delay in the transportation and could compromise the pulp quality, the pilot trial was carried out with washed O_2_ delignified eucalyptus kraft pulp, obtained directly from the mill (Portugal). The pulp showed 57% ISO brightness, kappa number of 11 and a drainage index of 18 °SR. About 10 kg of pulp was collected after D_1_ in order to compare the pulp quality after the four stages of the bleaching sequence (EXZ, D_0_, E_p_, D_1_). The pilot trial was stopped at D_1,_ since the desired brightness had been reached, but a final D_2_ stage was carried out at laboratory scale to see to which level the pulp could be bleached.

The CTP bleaching pilot plant is equipped with a specific reactor, which allows good reproducibility of an industrial bleaching stage, a twin wire press to perform washing and to prepare the pulp for the subsequent bleaching stage, pumps and mixers. At the end of the bleaching sequence, the twin wire press allows pulp sheets to be produced at ≈30% consistency.

### 3.7. Evaluation of Pulp Properties

For all experiments, pulp brightness was measured according to ISO 3688, 1999 and ISO 2470, 2016 standards and compared to the mill reference target. The standard deviation of all brightness measurements varied from 0.1 to 0.3% ISO.

Bleached pulps refined at different refining levels (0, 2000 and 3000 PFI revolutions according to ISO 5264-2 standard) were analysed. The papermaking properties were evaluated according to the standard procedures shown in Table S4. The acceptance of the replicates analysed for each sample are according to the standards presented in Table S4, which consider specific acceptance of error limits in each parameter. The mean values used to build Figure 5 and Figure 7 were computed using only those replicates within the acceptance limits (Table S1). Therefore, all the values presented are accurate according to the demands of the international standards.

### 3.8. Analysis of the Effluents

COD, BOD and AOX content of the bleaching effluents were, respectively, determined according to ISO 15705:2002, NF EN ISO 5815-1 and NF EN 1485 standards.

## 4. Conclusions

A novel bacterial xylanase with outstanding alkaliphilic properties and thermophilicity was successfully overproduced in *E. coli* and used as a prebleaching stage for ECF bleaching of O_2_-delignified hardwood kraft pulps.

The novel Xyn11-assisted bleaching sequence outperformed the bleaching efficiency of the industrial process. A pilot-scale validation of the extremozyme-assisted sequence on eucalyptus kraft pulp under relevant industrial conditions indicated that 25% ClO_2_ savings are made possible by removing the last D_2_ stage, while maintaining even higher pulp brightness than the mill reference, and with no detrimental effect on the pulp mechanical properties. Moreover, cellulose is less degraded during the D stages and lignin is more selectively extracted. Additional benefits from the reduction of the amount of ClO_2_ added in each D stage are the significant decrease in AOX content and a higher biodegradability of the bleaching effluent.

These excellent results were confirmed on a mixed hardwoods kraft pulp, obtaining similar ClO_2_ savings, and proving that the enzymatic stage could be directly applied at industrial level without major changes to the current pulp production process.

Finally, for the successful market introduction of a novel methodology or technology for a more sustainable production, technoeconomic performance must be validated. In the case of Xyn11, pilot scale (400 L) production demonstration already indicates a robust process, and the enzyme cost is expected to be at least in line with similar types of enzymes in the market, or even lower. This will enable lower barrier to market for this specific enzyme.

## Figures and Tables

**Table 1:** Final properties of the O_2_-Delignified eucalyptus kraft pulp (after D_2_ bleaching stage) for the enzymatic sequence (EXZD_0_EpD_1_D_2_) using less 15% ClO_2_, and for the reference sequence (D_0_EpD_1_D_2_) with ClO_2_ reference loading.

BLEACHING SEQUENCE	EXZD_0_E_p_D_1_D_2_	D_0_E_p_D_1_D_2_
Kappa Number (after Ep)	2.8	3.4
Pulp Brightness (% ISO)	91.1	89.0
Cellulose DPv	1240	1120
Drainage Index (°SR)	23	19
ClO_2_ Consumption (%)	91	100

## Supplementary Materials

The following supporting information can be downloaded at: https://www.mdpi.com/article/10.3390/ijms232113423/s1.
