# Supplementary material for: Use of a Novel Extremophilic Xylanase for an Environmentally Friendly Industrial Bleaching of Kraft Pulps
Source: Int J Mol Sci. 2022 Nov 3;23(21):13423. doi: 10.3390/ijms232113423 (PMC9654485; doi:10.3390/ijms232113423)
Supplement: Supplementary file 1 [file ijms-23-13423-s001.zip › ijms-1995351-supplementary.pdf]

Supplementary Material

Results

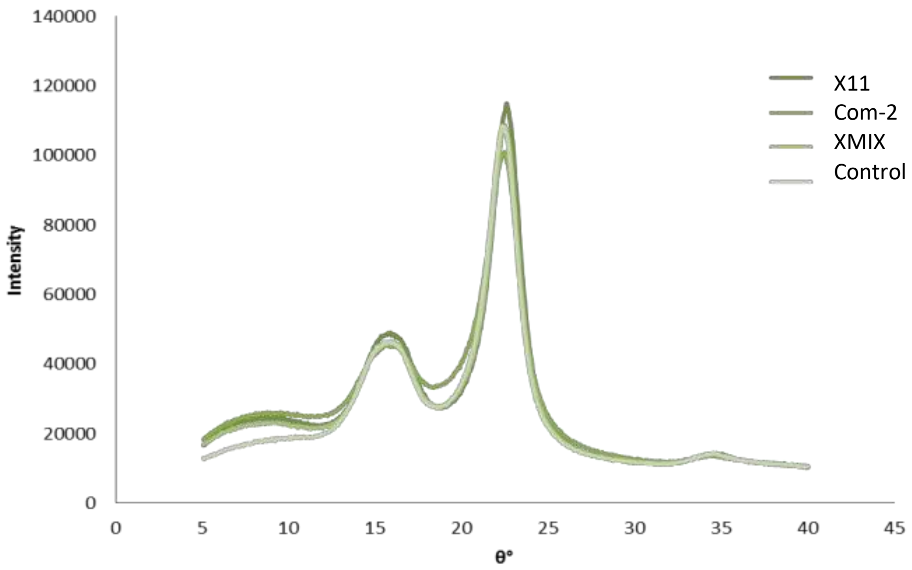

Figure S1. X-ray diffraction spectra of the pulps treated with the three xylanases (Xyn 11, Com-2, XMIX) compared to the control pulp.

Table S1. Crystallinity values for the pulps treated with the three xylanases (Xyn 11, Com-2, XMIX) compared to the control pulp.

Enzyme tested	Crystallinity index of the treated pulp (%)
X11	76.3
Com-2	67.2
XMIX	74.9
Control	74.8

Table S2. Analysis of the effluents generated by the Xyn11-assited bleaching ($XD_0E_pD_1$) of eucalyptus kraft pulp at pilot scale compared with the reference sequence ($D_0E_pD_1D_2$).

Bleaching sequence	X D Ep D	D Ep D D
**COD, kg/T of o.d. pulp**		
After X	8.3	-
After D_0	5.4	16.0
After Ep	11.9	23.4
After D_1	1.9	7.6
After D_2	-	2.6
**BOD, kg/T of o.d. pulp**		
After X	2.1	
After D_0	1.6	2.1
After Ep	5.1	5.7
After D_1	0.9	0.6
After D_2	-	0.04
**AOX, kg/T of o.d. pulp**		
After X	0	-
After D_0	0.15	0.16
After Ep	0.03	0.03
After D_1	0.04	0.05
After D_2	-	0.02

Material and Methods

Table S3. Medium composition for production of Xyn 11 at pilot scale in *E. coli.*

Component	g/L
CSL	15
Yeast extract	10
$(NH_4)_2SO_4$	0.4
KH_2PO_4	2.66
$MgSO_4$ x $7H_2O$	0.24
$K2HPO_4$	1.06
Citric acid	0.34
Antifoam	0.2
Technical water	Adjusted for start of fermentation volume

Table S4. Standards used for the determination of the papermaking properties.

Papermaking Property	Determination Procedure	Limit for acceptance (%)
Drainage index, Schopper-Riegler	ISO 5267-1:1999	±4%
Air Resistance – Gurley method	ISO 5636-5:2013	±20%
Burst Index	ISO 2758:2015	± 15%
Tensile Index	ISO 1924-2:2008	± 12%
Tear Index	ISO 1974:2012	± 9%
Scott Test	TAPPI 569	± 6%
